# Impaired HSP70 Expression in the Aorta of Female Rats: A Novel Insight Into Sex-Specific Differences in Vascular Function

**DOI:** 10.3389/fphys.2021.666696

**Published:** 2021-04-22

**Authors:** Amanda Almeida de Oliveira, Fernanda Priviero, R. Clinton Webb, Kenia Pedrosa Nunes

**Affiliations:** ^1^Laboratory of Vascular Physiology, Department of Biomedical and Chemical Engineering and Sciences, Florida Institute of Technology, Melbourne, FL, United States; ^2^Department of Physiology, Augusta University, Augusta, GA, United States; ^3^Department of Cell Biology and Anatomy, Cardiovascular Translational Research Center, University of South Carolina, Columbia, SC, United States

**Keywords:** Hsp70, sex differences, vascular contraction, vascular relaxation, calcium, nitric oxide, ROS

## Abstract

Heat-shock protein 70 (HSP70) contributes to cellular calcium (Ca^2+^) handling mechanisms during receptor-mediated vascular contraction. Interestingly, previous studies have independently reported sex-related differences in HSP70 expression and Ca^2+^ dynamics. Still, it is unknown if sex, as a variable, plays a role in the impact that HSP70 has upon vascular contraction. To narrow this gap, we investigated if differences exist in the expression levels of HSP70 in the aorta, and if targeting this protein contributes to sex disparity in vascular responses. We report that, compared with male animals, female rats present a reduction in the basal levels of HSP70. More compelling, we found that the blockade of HSP70 has a greater impact on phenylephrine-induced phasic and tonic vascular contraction in female animals. In fact, it seems that the inhibition of HSP70 significantly affects vascular Ca^2+^ handling mechanisms in females, which could be associated with the fact that these animals have impaired HSP70 expression. Corroborating this idea, we uncovered that the higher sensitivity of female rats to HSP70 inhibition does not involve an increase in NO-dependent vasodilation nor a decrease in vascular oxidative stress. In summary, our findings reveal a novel mechanism associated with sex-specific differences in vascular responses to α-1 adrenergic stimulation, which might contribute to unraveling the network of intertwined pathways conferring female protection to (cardio)vascular diseases.

## 1. Introduction

Sex differences in cardiovascular function and dysfunction are noted in different animal models, and more importantly, cardiovascular diseases, a worldwide leading cause of death, have a higher prevalence in men and post-menopausal women, indicating a protective role for female sex hormones in this system (for review, see Regitz-Zagrosek and Kararigas, 2017). While previous research revealed a plethora of pathways involved in this process, the precise molecular mechanisms responsible for sex-related differences in cardiovascular function and dysfunction are mostly unclear. The stress-induced molecular chaperone, Heat-shock protein 70 (HSP70), is well-known for its pivotal protein homeostasis function (Radons, 2016). Recently, we uncovered that this protein is also key for adequate vascular reactivity (de Oliveira and Nunes, 2020), a physiological mechanism linked to systemic blood pressure regulation (Brozovich et al., 2016). The interaction between HSP70 and vascular function occurs, at least in part, because this protein impacts calcium (Ca^2+^) handling mechanisms (de Oliveira et al., 2021), and the presence of Ca^2+^ determines the magnitude of arterial contraction. Interestingly, previous studies have reported sex-related differences in Ca^2+^ signaling under (patho)physiological conditions. Specifically, it has been shown that female rats have diminished Ca^2+^ influx (Crews et al., 1999; Barron et al., 2002; Thompson and Khalil, 2003), which might be attributed to the fact that they also have reduced levels of the STIM1/Orai1 complex compared with male animals (Giachini et al., 2012). However, it is unknown if the variable sex plays a role in the impact that HSP70 has upon vascular contraction.

The female heart has twice as much HSP70 than the average male counterpart, and it seems that the enhanced expression of HSP70 in this tissue contributes to cardioprotection, which can be prevented by ovariectomy (Voss et al., 2003). Furthermore, the heart is not the only tissue previously reported to have sex disparity in HSP70 expression. Differences have also been noted in the kidney and slow-twitch skeletal muscle (Voss et al., 2003). Additionally, similar levels of this protein were observed in the liver and fast-twitch skeletal muscle. In the latter, even though the basal levels of HSP70 in females are equivalent to the ones of males, this tissue presents a reduction in the capacity of inducing this protein in response to exercise (Paroo et al., 2002). In this sense, it seems that sex exerts a critical role in determining the tissue rate of production of HSP70, but unfortunately, to the best of our knowledge, experimental data looking for sex-specific changes in HSP70 expression in the aorta is still lacking. Changes in the expression levels of HSP70 associate with alterations in vascular responses to α-1 adrenergic stimulation in the aorta (Kim et al., 2004, 2007; de Oliveira and Nunes, 2020). Female rats have reduced force development during receptor-mediated contraction in this blood vessel, which occurs in intact and endothelium-denuded arteries (Robert et al., 2005), but whether this response is linked to HSP70 is unknown. Therefore, we designed this study to establish if differences exist in the basal expression levels of HSP70 in the aorta as well as if targeting this protein contributes to sex disparity in vascular response in this tissue.

## 2. Materials and Methods

### 2.1. Chemicals and Solutions

All chemicals used in this study were purchased from Millipore-Sigma (St. Louis, MO, United States), unless stated otherwise. Stock solutions were initially diluted in Dimethyl Sulfoxide (1 mol/L; N^o^ D2650) and kept at −20°C. Working solutions were freshly prepared right before the experiment by diluting stock solutions in physiological salt solution (PSS); therefore, PSS was considered to be the vehicle in all experiments.

### 2.2. Animals

Twenty-four Sprague Dawley rats, equally divided between sexes, were acquired from Taconic Biosciences and Envigo. Animals were kept in a controlled environment (temperature: 22 ± 1°C; humidity: 42 ± 2%) with unrestricted access to food and tap water and were killed by exsanguination at 3–4 months of age under isoflurane anesthesia (5% in 100% O_2_). Body mass and glucose levels were determined right before euthanizing the animal. The latter was determined using a commercially available blood glucose monitoring system in non-fasted animals. From each rat, we excised the thoracic aorta, which was placed in cold PSS (mmol/L: 130 NaCl, 4.7 KCl, 1.18 KH_2_PO_4_, 1.18 MgSO_4_.7H_2_O, 14.9 NaHCO_3_, 5.6 Dextrose, 1.56 CaCl_2_.H_2_O, 0.026 EDTA). Then the vessel was cleaned of fat tissue and further processed according to the specific experimental protocol.

### 2.3. Western Blotting

The expression levels of HSP70 were determined in the aorta of male and female animals. Briefly, the aorta was homogenized in extraction buffer—mixture of Tissue Protein Extraction Reagent (ThermoFisher Scientific, N^o^ 78510) and a Protease Inhibitor Cocktail (N^o^ P8340)—and stored at −80°C. Total protein concentration was determined with a BCA Protein Assay kit (ThermoFisher Scientific, N^o^ 23225). A total of 15 μg of protein was loaded into a 10% SDS-PAGE gel and transferred to a nitrocellulose membrane. Non-specific binding sites were blocked with 5% non-fat-dry milk diluted in Tris-buffered solution with 1% Tween for 1 h at room temperature. The membrane was then probed overnight at 4°C with primary antibody (Cell Signaling, N^o^ 4872, 1:1,000) diluted in Tris-buffered solution with 1% Tween + 0.5 g of bovine serum albumin. The secondary antibody (Cell Signaling, N^o^ 7074, 1:10,000) was allowed to react with the membrane for 1 h at room temperature under constant agitation. Immunoblots were revealed by the SuperSignal West Femto Substrate (Thermo Fisher Scientific, N^o^ 34095) using automatic exposure time in the Chemidoc MP Imaging System (Bio Rad, California, USA). Protein levels were quantitated using the ImageJ software (NIH, Bethesda, MD, USA) and normalized to β-actin (Santa Cruz Biotechnology, N^o^ sc-47778, 1:1,000) expression.

### 2.4. Functional Studies

The thoracic aorta was cut into 2 mm rings, which were mounted in a DMT620M multi wire myograph system (Danish Myo Technology, Aarhus, Denmark) with a resting tension of 15 mN/mm. The chambers were filled with 5 ml of PSS and gassed with carbogen (95% CO_2_ and 5% O_2_, 37°C) to maintain a pH of 7.4. Then, samples were allowed to reach an equilibrium state (~1 h). The viability of the preparation was tested with a high KCl (120 mmol/L) solution, which was prepared as a modified PSS where the concentration of NaCl was equimolar to KCl. Viable samples, which were the ones that, following the addition of the KCl solution, had a force displacement >50% of the preload, were washed and allowed to return to the resting tension (around 30 min). The presence of the endothelium was determined by the ability of acetylcholine (10^−5^ mol/L) to induce relaxation in arteries pre-contracted with phenylephrine (10^−5^ mol/L) to achieve a contractile response of ~50% of the KCl contraction (Garćıa-Redondo et al., 2018). Then, aortic rings were incubated with vehicle or VER155008 (10^−5^ mol/L, N^o^ SML0271) for 30 min. Of note, VER155008 is a small molecule inhibitor that selectively targets HSP70 via its ATPase domain (Massey et al., 2010). Subsequently, samples were challenged with a single dose of phenylephrine (10^−5^ mol/L), and a concentration response curve to acetylcholine (10^-9^ − 10^-4^ mol/L) or clonidine (10^-9^ − 10^-4^ mol/L) was obtained.

In another set of experiments, a new batch of endothelium-intact aortic rings were processed as mentioned above until the viability test. Thereafter, we replaced the PSS in the myograph chamber with a Ca^2+^ free solution supplemented with 1 mmol/L EGTA. Then, after 3 min, we stimulated the samples with a single dose of phenylephrine (10^−5^ mol/L). The force developed was evaluated for 10 min. Next, we restored the initial Ca^2+^ concentration to the solution, and we recorded the force generated for 15 min. Experiments were conducted in vehicle and VER155008 (10^−5^ mol/L)-treated samples.

#### 2.4.1. Data Analysis

The values of relaxation were computed as % of the maximal contraction elicited by the agonist, which was considered 100% in all cases. The relationship between time (T) and force (F) expressed in the time-force (s-mN) curves was calculated in the following way. We considered the moment we added phenylephrine (10^−5^ mol/L) into the chamber as *t*_0_. At any point in time after *t*_0_, *F* was calculated by subtracting the basal force, which is the observed measurement *F* at *t*_0_. Curves were analyzed for 600 s with *F* being sampled every second. Then, we calculated the amplitude of the phasic (*A*_phasic_) and tonic (*A*_tonic_) parts of the contraction curve. The former was computed by subtracting the *F* observed at *t*_phasic_ of the basal force, and the latter was determined by subtracting the *F* observed at *t*_tonic_ of the *A*_phasic_ part of the curve. We considered *t*_phasic_ as the moment the vessel shifted from a rapid force development pattern to a slow phenotype, which was not consistent between animals, and *t*_tonic_ as 600 s, which was the last time point sampled in the curve. Subsequently, we assessed the % inhibition of phenylephrine-induced contraction in relation to samples incubated with only vehicle, which was taken as 100% in all cases. The time-force curves in the Ca^2+^ protocol was constructed as mentioned above, except that we considered the moment we added the Ca^2+^ free PSS into the chamber as t_0_.

### 2.5. *In situ* Detection of Nitric Oxide and Reactive Oxygen Species

The thoracic aorta was cut into 2–3 mm rings, incubated with vehicle or VER155008 (10^−5^ mol/L) for 6 h in an isolated muscle bath bubbled with carbogen (95% CO_2_ and 5% O_2_, 37°C), and subsequently frozen in OCT compound. Aortic rings were not subjected to a resting tension. Next, we obtained transverse sections of the rings (10 μm) in a cryotome. Then, the slides were incubated with fluorescent probes to target nitric oxide (DAF-FM, ThermoFisher Scientific, N^o^ D23844, 10 μmol/L diluted in PBS) and reactive oxygen species (DHE and H_2_DCFDA, ThermoFisher Scientific, N^o^ D11347 and D399, 10 μmol/L diluted in PBS) protected from light for 30 min. To make sure that the fluorescent signal observed in samples incubated with DAF-FM was due to nitric oxide levels and not to elastin autofluorescence, we imaged sections, collected under the same conditions, but in the absence of DAF-FM. Additionally, L-NAME (10^−6^ mol/L; N^o^ N5751, 30 min), a selective NOS inhibitor, was used as a negative control. Some aortic slices were incubated with a cell permeant superoxide dismutase mimetic, MnTMPyP (25 μmol/L; Fisher Scientific, N^o^ 50-200-9179), for 30 min at 37°C, to control for the specificity of the DHE probe. When using the DHE probe, diethylenetriaminepentaacetic acid (100 μmol/L, N^o^ D6518) was applied to each section. Images were acquired with a 20 × objective lens with equal exposure time for all samples on a Zeiss Observer A1 AXIO inverted microscope. Digital images were collected using Photometrics CoolSNAP MYO camera. The fluorescence intensity was measured using the freely available software imageJ in images with the background removed (NIH, Bethesda, MD, USA), and it is displayed as arbitrary units. Representative images from all groups were modified for brightness and contrast using the same parameters throughout the whole image.

### 2.6. Statistical Analysis

Results are reported as mean ± standard error of the mean (SEM) and the letter *n* denotes the number of animals used in that set of experiments. Statistical differences between groups in the concentration-response curves were determined using two-way ANOVA. For the time-force and Ca^2+^ protocol curves, one-way ANOVA was used. In both cases, the Bonferroni correction was applied to counteract the problem of multiple comparisons. In all other analysis reported in this manuscript, statistical differences between groups were verified using a Student's two tailed *t*-test. All statistical analysis were conducted using the software GraphPad Prism (version 6.0) and a *p* ≤ 0.05 was considered statistically significant.

## 3. Results

### 3.1. HSP70 Expression in the Aorta: Does Sex Matter?

**Figure 1:**
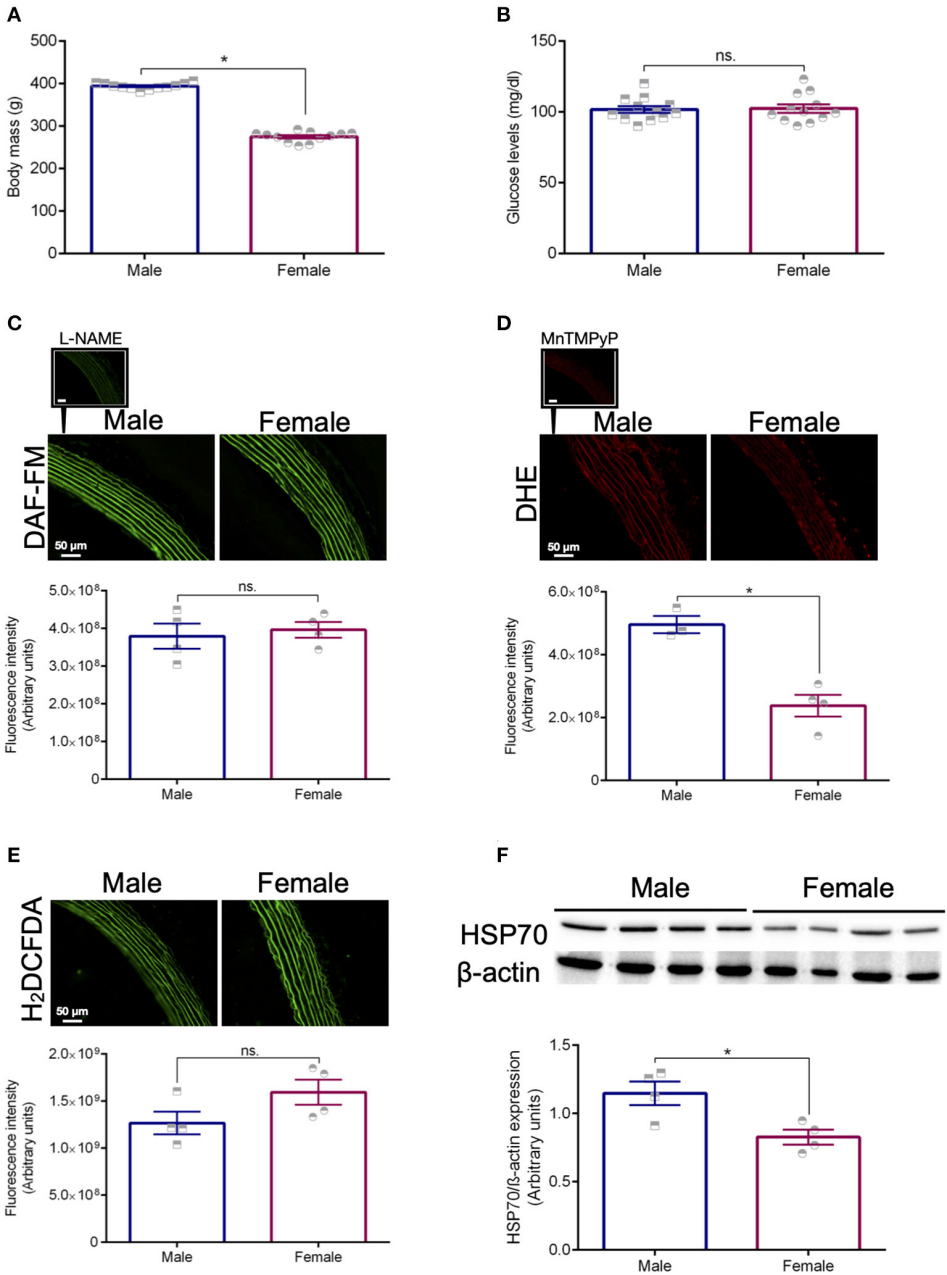
Animal profile. Body mass **(A)** and glucose levels **(B)**. The fluorescent probes DAF-FM **(C)**, DHE **(D)**, and H_2_DCFDA **(E)** were used to measure the levels of nitric oxide and vascular oxidative stress in isolated thoracic aortas from male and female Sprague Dawley rats. L-NAME (10^-6^ mol/L, NOS inhibitor) was used as a negative control for DAF-FM and MnTMPyp (25 μmol/L, superoxide dismutase mimetic) was applied to control for the specificity of the DHE probe. Expression levels of HSP70 after normalization to β-actin **(F)**. Data are expressed as mean ± SEM, *n* = 4 except for the male group in **(D)** (*n* = 3). *p ≤ 0.05 using two-tailed Student's *t*-test.

The female rats used in this study presented lower body mass and similar glucose levels compared with age-matched male animals (Figures 1A,B, respectively). Differences were noted between sexes for the basal levels of superoxide (Figure 1D), but not for nitric oxide (Figure 1C). Additionally, no variations were detected with the fluorescent probe H_2_DCFDA (Figure 1E). Following this initial characterization, we assessed the expression levels of HSP70 in the aorta of male and female animals using standard Western blotting technique. We found that, compared with male rats, female animals have significantly lower basal levels of this protein (Figure 1F), which might be a mechanism affecting the contraction pattern of this vascular structure.

### 3.2. Comparing the Impact of Blocking HSP70 in Male and Female Animals

#### 3.2.1. Vascular Contraction

**Figure 2:**
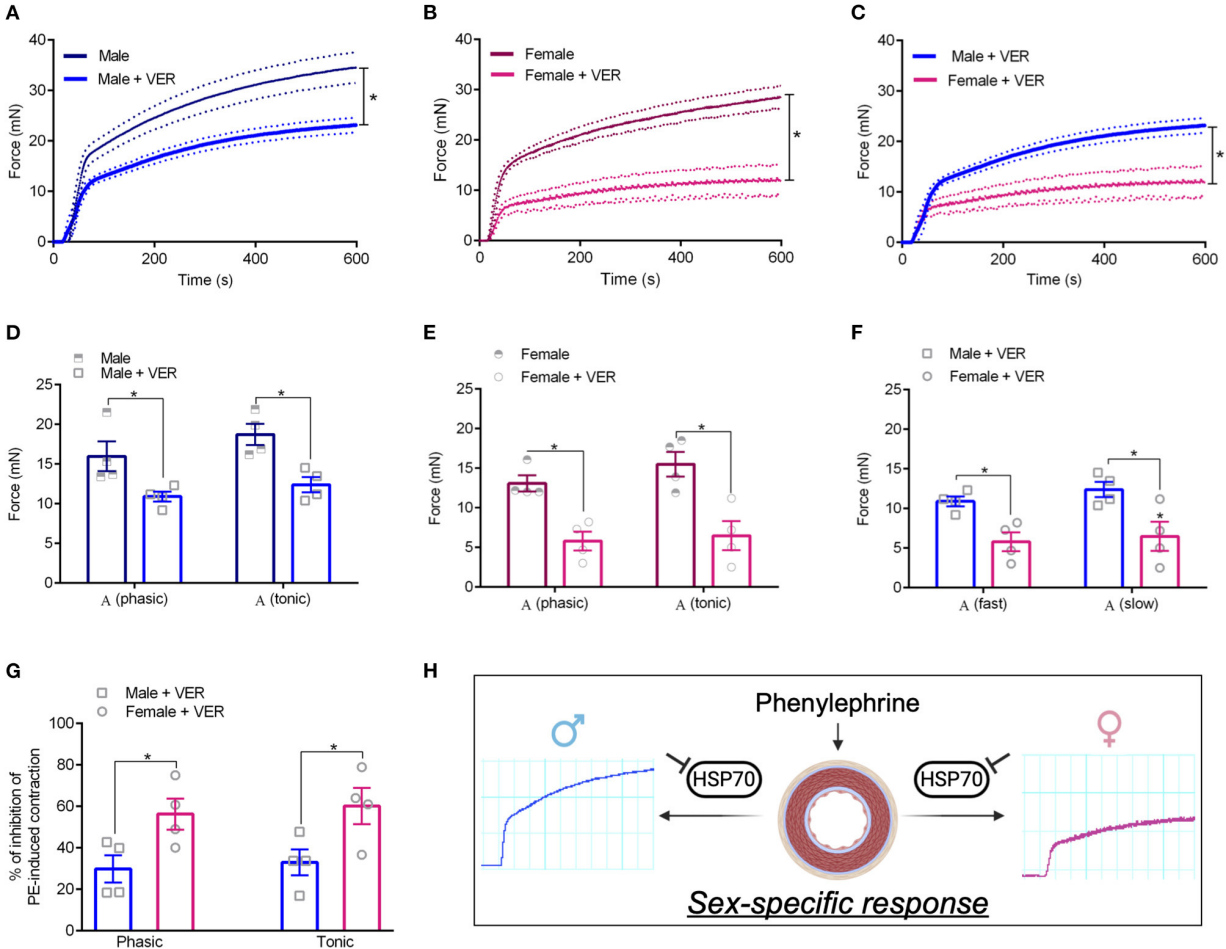
Blockade of HSP70 significantly impairs phasic and tonic vascular contraction in female compared with male rats. **(A–C)** Display time vs. force curves for aortic samples incubated with vehicle (CTL) or VER1550008 (10^−5^ mol/L) for 30 min and stimulated with phenylephrine (10^−5^ mol/L) for 15 min. *p ≤ 0.05 using one-way ANOVA followed by the Bonferroni correction. Phasic and tonic E_max_ in male **(D)**, female **(E)**, and male vs. female **(F)**. % of inhibition of phenylephrine-induced contraction in male vs. female **(G)**. *p ≤ 0.05 using two-tailed Student's *t*-test. Schematic representation with inset original trace recording graphs showing the vascular response to phenylephrine following inhibition of HSP70 in male and female Sprague Dawley rats **(H)**. **(H)** Was created with Biorender.com. Data points were sampled every second for 600 s and are expressed as mean ± SEM, where the continuous line represents the mean and the traced lines depict the SEM. *n* = 4.

We previously demonstrated that the *ex vivo* inhibition of HSP70 with VER155008 (10^−5^ mol/L, 30 min) impairs phenylephrine-induced phasic and tonic contraction in male animals (de Oliveira and Nunes, 2020), which was replicated in this study (Figures 2A,D). To build on this finding, we next evaluated if pharmacological blockade of HSP70 also impacts vascular contraction in response to α-1 adrenergic stimulation in female animals. Here, we observed that, in these animals, the inhibition of HSP70 leads to a profound decrease in the maximum contraction elicited by the agonist in both phases (Figures 2B,E). More interesting, the impairment of the response appeared to be greater in female rats than male animals (Figures 2C,F). Here, we acknowledge that, under this experimental condition, female animals have a reduced ability to elicit contraction. Therefore, we also calculated the % of inhibition of phenylephrine-induced contraction, and we confirmed that the blockade of HSP70 significantly impairs phasic and tonic vascular contraction in female compared with male rats (Figure 2G), which led us to propose that the variable sex is key for determining the impact that HSP70 inhibition has upon the contraction phenotype of isolated aortas (Figure 2H).

**Figure 3:**
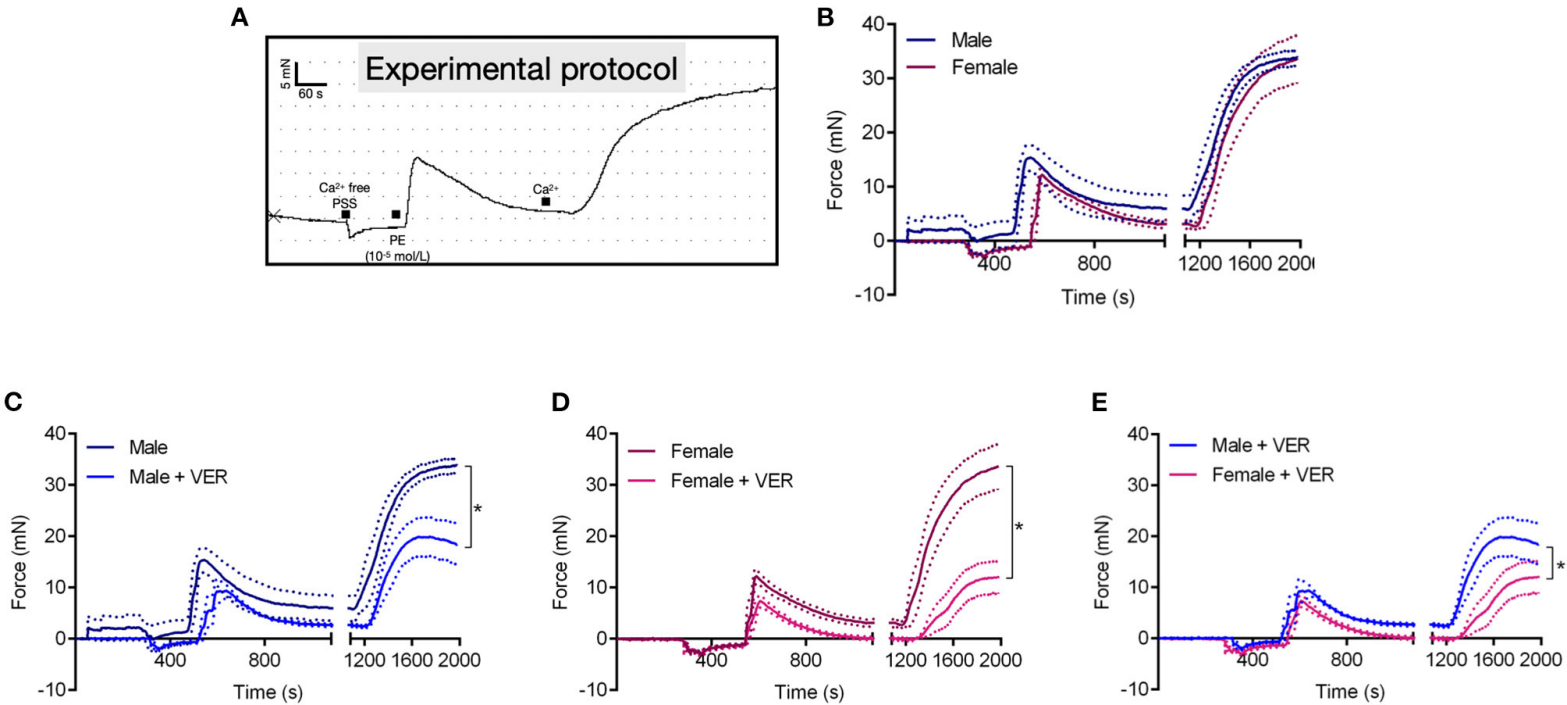
Blockade of HSP70 greatly affects Ca^2+^ influx-mediated vascular contraction in female compared with male rats. Schematic of the protocol used in this set of experiments **(A)**. Male and female aortic rings were stimulated with phenylephrine (10^−5^ mol/L) in Ca^2+^ free physiological salt solution for 10 min. Subsequently, the extracellular concentration of Ca^2+^ was restored and the force developed was analyzed for 15 min **(B)**. **(C–E)** Show the data for male, female, and male vs. female, respectively in the presence of vehicle or VER155008 (10^−5^ mol/L). Data points were sampled every second for 1,980 s and are expressed as mean ± SEM, where the continuous line represents the mean and the traced lines depict the SEM. *n* = 4, *p ≤ 0.05 using one-way ANOVA followed by the Bonferroni correction.

Previous studies have demonstrated sex-related differences in Ca^2+^ signaling, which contributes to differences between sexes in vascular contraction (Crews et al., 1999; Barron et al., 2002; Giachini et al., 2012). Recently, we reported that HSP70 contributes to Ca^2+^ handling mechanisms during receptor-mediated contraction in male animals (de Oliveira et al., 2021). Considering this previous knowledge and our above-mentioned findings, we decided to investigate if the mechanism by which HSP70 greatly affects vascular contraction in females compared with males involves vascular Ca^2+^ dynamics (Figure 3A). We found that male and female animals present a similar contraction pattern in Ca^2+^ free PSS (Figure 3B), which is consistent with previous studies (Murphy and Khalil, 2000; Fransen et al., 2015). As expected, blockade of HSP70 reduced the magnitude of the contraction following the re-addition of Ca^2+^ in both sexes (Figures 3C,D). More interestingly, was the observation that the blockade of HSP70 substantially affected Ca^2+^ influx-mediated vascular contraction in female compared with male rats (Figure 3E).

#### 3.2.2. Vascular Relaxation

**Figure 4:**
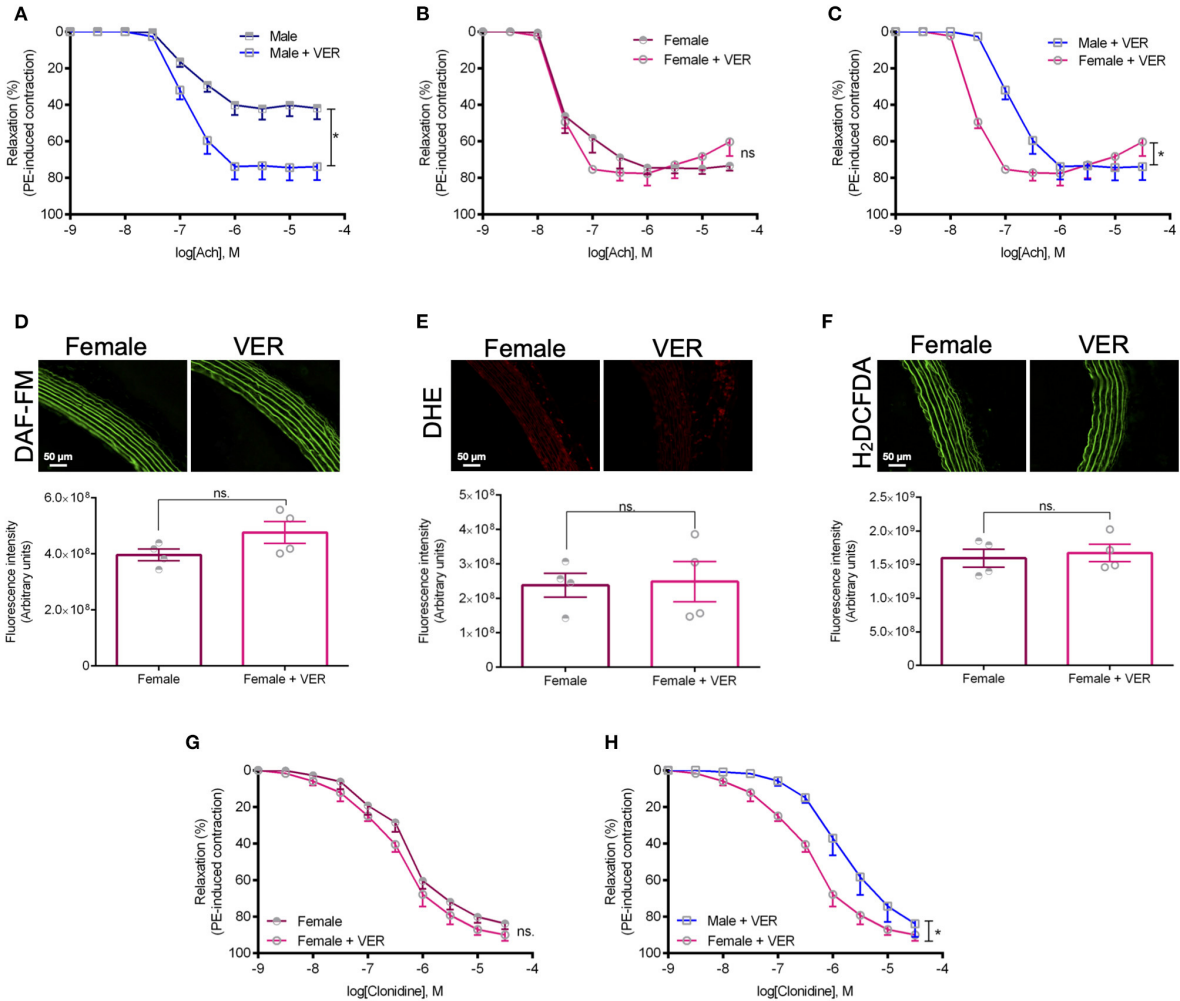
The higher sensitivity of female rats to HSP70 inhibition does not involve an increase in NO-dependent vasodilation nor a decrease in vascular oxidative stress. **(A–C)** Show concentration-response curves to acetylcholine and **(G,H)** display concentration-response curves to clonidine. *p ≤ 0.05 using two-way ANOVA followed by the Bonferroni correction. The fluorescent probes DAF-FM **(D)**, DHE **(E)**, and H_2_DCFDA **(F)** were used to measure the levels of nitric oxide and vascular oxidative stress in isolated thoracic aorta from female rats incubated with vehicle or VER155008 (10^−5^ mol/L), and are displayed as arbitrary units. *p ≤ 0.05 using two-tailed Student's *t*-test. Data are expressed as mean ± SEM, *n* = 4.

To gain additional mechanistic insights into the underlying pathways contributing to this response, we next investigated whether the inhibition of HSP70 would also facilitate endothelium-dependent relaxation in aortas isolated from female animals. Here, we focused on the female sex as we had previously demonstrated that rings isolated from male animals display an augmented relaxation response to acetylcholine in the presence of an inhibitor for HSP70 (de Oliveira and Nunes, 2020), which was confirmed in this work (Figure 4A). To our surprise, we did not find any differences between the female vehicle and VER155008-treated groups (Figure 4B), which, again, reinforces the notion that differences exist between the male and female response following the modulation of HSP70 (Figure 4C). Since the response elicited by acetylcholine depends on the integrity of the endothelium to generate nitric oxide, we also verified the levels of this gaseous transmitter. We observed that the HSP70 inhibitor did not affect nitric oxide levels in female rings (Figure 4D), similar to what happens in male isolated aortas (de Oliveira and Nunes, 2020). Corroborating these data, we also confirmed that the levels of vascular oxidative stress were unaltered in the presence of the HSP70 inhibitor, both in female (Figures 4E,F) and male (de Oliveira and Nunes, 2020) animals. A similar relaxation pattern was also detected when comparing female responses to clonidine, an α-2 adrenergic agonist, in the presence or absence of the HSP70 inhibitor (Figure 4G) as well as when comparing the relaxation pattern of male and female rings following the blockade of this protein (Figure 4H).

## 4. Discussion

HSP70 is a widespread molecular chaperone with emergent roles in vascular biology. In fact, we previously demonstrated that this protein assists in vascular contraction by affecting Ca^2+^ handling mechanisms (de Oliveira and Nunes, 2020; de Oliveira et al., 2021). In this work, we expand the concept that HSP70 exerts a key function in the vasculature as we show, for the first time, the contribution of this protein for the contraction phenotype of female isolated aortas. Here, it is relevant to mention some pitfalls of our study, such as the fact that it lacks female hormonal cycle control as well as that we only demonstrate this feature in a large conducting artery. However, it has been recently suggested that vascular dysfunction in large arteries impairs the vessels' compliance, elevating the conduction of pulsatility to the microcirculation (Climie et al., 2019; Schiffrin, 2020), which justifies the conduction of functional studies in the aorta. Sex-specific differences in vascular function occur under physiological conditions (Barron et al., 2002; Thompson and Khalil, 2003; Robert et al., 2005; Giachini et al., 2012), and more importantly, the variable sex plays a central role in the development of cardiovascular diseases (Kander et al., 2017; Regitz-Zagrosek and Kararigas, 2017). Additionally, previous studies link HSP70 to the pathophysiology of cardiovascular/renal diseases (Cai et al., 2010; Pons et al., 2013; de Oliveira et al., 2019a,b; Liu et al., 2019). Therefore, our findings are of utmost importance collectively corroborating the notion that HSP70 might be a novel insight into sex-specific differences in vascular function, and ultimately, opening research avenues for future work conducted in a disease state setting.

Specifically, we observed that, compared with male controls, female rats have reduced levels of HSP70 in the aorta (Figure 1F). As we mentioned above, previous studies have also shown a relationship between the variable sex and the expression levels of HSP70 in other tissues (Voss et al., 2003). Here, we believe that the impairment in HSP70 expression observed in female rats could help explain why these animals display higher sensitivity to the inhibition of this protein (Figure 2G). If we consider that previous studies demonstrated that fluctuations in the expression levels of HSP70 directly associate with the magnitude of contraction (Kim et al., 2004, 2007; de Oliveira and Nunes, 2020), it is reasonable to assume that the impaired levels of HSP70 in female animals are a factor contributing to reduced force generation following α-1 adrenergic stimulation. More precisely, when we block HSP70, because female animals already have reduced levels of this protein and lower force development, this phenomenon might be exacerbated with less HSP70 available to assist in the contraction mechanism. A previous study has suggested that aortic rings exposed to heat-stress, which predominantly induces the expression of HSP70, have increased vascular contraction due to modulation of thick filament, a mechanism dependent upon augmented levels of intracellular Ca^2+^ requiring phosphorylation of regulatory myosin light chain (Kim et al., 2004). However, it has been shown that unlike small HSPs, HSP70 does not directly affect the actin-myosin complex (Frossard et al., 2005). In fact, HSP70 appears to modulate vascular contraction trough an interaction with Ca^2+^ handling mechanisms (de Oliveira et al., 2021). Strengthening this argument, even though in the absence of external Ca^2+^ no differences are observed between sexes in the contraction phenotype of vascular cells (Murphy and Khalil, 2000; Fransen et al., 2015), female animals also presented higher sensitivity to HSP70 inhibition in the protocol designed to investigate Ca^2+^ influx-mediated vascular contraction (Figure 3E). This pattern could be due to the fact that HSP70 contributes to Ca^2+^ influx-mediated contraction by interacting with voltage-independent channels (de Oliveira et al., 2021), and in comparison with male rats, female animals were reported to have lower expression levels of these receptors (Giachini et al., 2012).

Our functional studies were conducted with a resting tension of 15 mN/mm, which is considered physiological preload since according to Laplace's Law it translates to a transmural pressure of 100 mm/Hg (De Moudt et al., 2017). This is relevant because, under the conditions evaluated in this study, intact aortic rings isolated from male rats displayed a relaxation response to acetylcholine of ~40% (Figure 4A), diverging from female rats (Figure 4B) as well as studies conducted under optimal preload. While it seems like male rings had a depressed response to acetylcholine, the work of De Moudt et al. (2017) warned about the extrapolation of functional data collected at an optimal preload, which might not reflect what is happening in the human physiology. From our data, it is also possible to infer that, unlike in male animals (Figure 4A), the blockade of HSP70 in female rats does not increase endothelium-dependent relaxation (Figure 4B), even though aortic rings incubated with the HSP70 inhibitor seem to reach maximum relaxation before the samples challenged with only vehicle. In this sense, one could argue that differences were not noted because female animals already display higher acetylcholine-mediated vasodilation. However, when we assessed NO production, we also did not observe augmentation in the generation of this gaseous transmitter (Figure 4D), which is the primary mediator of vasorelaxation in the presence of acetylcholine (Furchgott, 1983; Chataigneau et al., 1999). Corroborating this argument, we also did not encounter changes in vascular oxidative stress (Figures 4E,F) using the fluorescent probes DHE and H_2_DCFDA. The former targets superoxide and the latter is a global indicator of ROS, commonly used to verify the levels of hydrogen peroxide (Yang et al., 2014), though not selectively (Dikalov and Harrison, 2014). It is also important to mention here that female animals presented lower levels of superoxide compared with male rats, but no changes were observed with the H_2_DCFDA probe (Figures 1D,E). This could be explained as female vascular smooth muscle cells have increased superoxide dismutase activity (Morales et al., 2015), which is the enzyme that catalyzes the dismutation of superoxide into hydrogen peroxide (Fukai and Ushio-Fukai, 2011), and as stated H_2_DCFDA might target this type of ROS. The data reported for NO and vascular ROS in female aortas accompany our findings with male samples (de Oliveira and Nunes, 2020), which strengthens the idea that, in male animals, the pharmacological inhibition of HSP70 does not increase vasodilation but instead acts as a facilitator of this physiological process. Regarding the fact that we observed that the HSP70 inhibitor acts as a facilitator of vascular relaxation in males, but not in females, it is also important to consider that there is still debate about the underlying mechanisms leading to sex-related differences in vascular relaxation; however, there is a consensus that putative alterations might exist between sexes. For example, the relaxation response to acetylcholine is more affected by NO inhibition in female than male animals, suggesting that other mechanisms might contribute to relaxation in males whereas females mainly rely upon NO release (Loria et al., 2014). This happened albeit the fact that the aorta of male and female rats have similar NOS activity, but whether there are alterations following acetylcholine stimulation, it is still elusive.

Here, we believe it is important to acknowledge some limitations of our work. First, we evaluated the impact of blocking HSP70 in rings isolated from male and female intact animals. It is imperative to consider the role of sex hormones, especially estrogen, the female sex hormone, in the outcomes measured. Even though a previous study has demonstrated that the vascular heat-shock response occurs independently of estrogen deprivation (Miragem et al., 2015), indicating that the aging process in females does not impair this putative mechanism, the effects of estrogen cannot be trivialized and further studies are still needed to rule out its impact. Second, while we used a well-established protocol to assess vascular contraction in response to Ca^2+^ influx in isolated aortas (Fransen et al., 2015; de Oliveira et al., 2021), we did not directly measure Ca^2+^ levels following α-1 adrenergic stimulation. Therefore, while we are tempted to tie our findings to the fact that (a) female animals have reduced expression of STIM1/Orai1, and consequently, lower Ca^2+^ influx (Crews et al., 1999; Giachini et al., 2012); and (b) based on our previous work this voltage-independent mechanism appear to be the one targeted by HSP70 (de Oliveira et al., 2021), we believe caution should be exercised when drawing conclusions from this set of analysis. Lastly, we performed experiments with the small molecule inhibitor VER155008, which acts by inhibiting the ATPase activity of HSP70 (Massey et al., 2010). In this case, we believe that future studies with samples overexpressing HSP70 could add necessary details about underlying mechanisms. Nevertheless, we strongly believe that our data fill in a knowledge gap by providing a potential new mechanism contributing to sex-related differences in vascular function.

Overall, we show that a functional HSP70 is also crucial for proper vascular reactivity in female animals. In fact, reduced levels of HSP70 in these animals are mostly like a factor contributing to lower force-generation during receptor-mediated contraction. From a broader perspective, our work leads to the formation of more complex questions, such as whether this intertwined relationship between HSP70 and vascular contraction could help explain why female animals are less susceptible to the development of hypercontractility as well as if the modulation of this protein could offer a target-mechanism in diseased states. Moving forward, studies are required to narrow these questions, which will hopefully contribute to the understanding of the network of pathways conferring female protection to (cardio)vascular diseases.

## Data Availability Statement

The original contributions presented in the study are included in the article/supplementary material, further inquiries can be directed to the corresponding author/s.

## Ethics Statement

The animal study was reviewed and approved by Institutional Animal Care and Use Committees of Augusta University and Florida Institute of Technology.

## Author Contributions

AAO and KPN conceived the experimental design. AAO, FP, and KPN conducted the experiments. AAO performed the data analysis and wrote the first draft of the manuscript. FP, RW, and KPN revised the manuscript for intellectual content. All authors read and approved the final version of the manuscript.

## Conflict of Interest

The authors declare that the research was conducted in the absence of any commercial or financial relationships that could be construed as a potential conflict of interest.
